# The role of MYEOV gene: a review and future directions

**DOI:** 10.3389/fonc.2025.1543590

**Published:** 2025-06-04

**Authors:** Yidan Xi, Xuefei Liu, Yanan Ge, Mingzhe Jiang, Dong Zhao, Ershu Zhao, Fei Cai, Xinlong Wang, Jiatong Li, Tingting Li, Zhendong Zheng

**Affiliations:** ^1^ Department of Oncology, General Hospital of Northern Theater Command, Shenyang, China; ^2^ Department of Periodontics, Stomatological Hospital, School of Stomatology, Southern Medical University, Guangzhou, China

**Keywords:** MYEOV, target, biomarker therapy, signaling pathway, tumor

## Abstract

The overexpressed gene MYEOV in multiple myeloma, as an oncogene, has been widely recognized for its high expression levels in various malignant tumors. MYEOV plays a significant role in multiple malignancies, particularly in diseases such as multiple myeloma, breast cancer, lung cancer, pancreatic cancer, and esophageal cancer. The presence of the open reading frame of MYEOV in humans and other primates suggests its potential protein-coding capacity, although direct evidence of functional MYEOV protein is currently lacking. The role of MYEOV in various tumors is not limited to its direct effects as an oncogene; it also includes its complex role in tumor cell signaling pathways and its ability to participate in miRNA regulatory networks as a competing endogenous RNA (ceRNA). Studies have shown that MYEOV may affect the expression of cancer-related genes through enhancer activity. This article aims to provide a comprehensive review of the role of MYEOV, its involvement in signaling pathways in tumor cells, and the latest advancements in MYEOV-targeted therapies.

## Introduction

Orphan genes refer to genes that are restricted in expression within specific lineages and lack similar sequences in other lineages. The expression of these genes is typically characterized by significant temporal and tissue specificity, playing a crucial role in a variety of biological processes such as embryonic development, tissue differentiation, rare diseases, and the occurrence and development of tumors (1). Among orphan genes, MYEOV is considered a potential proto-oncogene, closely associated with gene rearrangement in various malignant tumors and correlated with poor prognosis in multiple types of cancer. The RNA expression levels of the MYEOV gene are closely related to the prognosis of cancer patients.

The MYEOV gene was initially isolated from the DNA samples of gastric cancer patients through the NIH/3T3 tumorigenicity assay and was found and activated in a subset of multiple myeloma cell lines with specific chromosomal translocations (t(11;14)(q13;q32)) (2). Studies suggest that during the differentiation process of primates, particularly during the divergence of Catarrhini/Platyrrhini, MYEOV may acquire a new open reading frame at the start codon of MYEOV-313 through mutation, potentially participating in protein coding. It is noteworthy that the protein-coding potential of MYEOV appears to be limited to humans and has not been found in other primates (3).

Within the professional domain, MYEOV (myeloma overexpressed gene) has been widely reported as an oncogene that is overexpressed in multiple myeloma (3). The latest research further reveals the phenomenon of dysregulated expression of MYEOV in the occurrence and progression of somatic tumors (4). MYEOV is involved in the pathogenesis of a variety of cancers, including multiple myeloma, neuroblastoma, esophageal cancer, breast cancer, lung cancer, gastric cancer, and colorectal cancer (5). Although the overexpression of MYEOV in cancer patients is associated with its oncogenic function, the underlying molecular mechanisms of the tumorigenic phenotype mediated by MYEOV have not been fully elucidated (6). Given the significant role of MYEOV in multiple types of cancer, it has emerged as a promising biomarker, offering new perspectives for the diagnosis and prognosis of cancer (4).

## The localization of MYEOV

Recent research has unveiled MYEOV as a novel transforming gene, initially discovered in the 11q13 chromosomal region in myeloma. This gene is frequently amplified in breast and esophageal cancer cell lines (5). Amplification of the 11q13 chromosomal region is prevalent in a variety of human cancers, including breast, bladder, esophageal, and head and neck tumors, as well as various hematological malignancies, such as the t(11;14)(q13;q32) translocation in B-cell neoplasia, particularly common in non-Hodgkin’s lymphoma and multiple myeloma (MM). This recurrent chromosomal alteration juxtaposes the CCND1 gene with the IgH-5 Eµ enhancer located at 14q34.2, leading to dysregulation of CCND1 gene expression, which is considered a key molecular event in the development of hematological malignancies with the t(11;14) (6). The human cancer gene hst (fgf4) is situated approximately 9kb away from the MYEOV gene. MYEOV and hst (fgf4) are typically located on the 11q13 band, about 475kb apart, and this region is often amplified and overexpressed in various tumors (7). In a subset of multiple myeloma (MM) cell lines with the t(11;14) and T, the presence of MYEOV may be related to the regulation of CCND1 by different IgH enhancers, leading to reciprocal translocations between them (8). Through DNA fiber fluorescence *in situ* hybridization (FISH) technology, MYEOV is localized to a 360kb centromeric region of cyclin D1. In all myeloma cell lines, translocation breakpoints are consistently localized within the 360kb region between MYEOV and cyclin D1 (2).

Researchers including Coccaro utilized fluorescence *in situ* hybridization (FISH) technology to conduct a precise localization study of the MYEOV gene, demonstrating that it is in close proximity to the CCND1 gene, with a total length of 360kb (8). In specific subsets of esophageal squamous cell carcinoma, breast cancer, gastric cancer, neuroblastoma, and colorectal cancer, the MYEOV gene is co-amplified and overexpressed with the CCND1 gene. However, the expression of MYEOV is detected only in cell lines harboring MYEOV gene amplification. The MYEOV gene is located 390kb upstream of the CCND1 gene, and its 5’ untranslated region (UTR) contains four upstream translation initiation sites that prevent the translation of the MYEOV gene in human cells. These four upstream AUG sequences regulate the ribosome entry site, preventing ribosome binding to the MYEOV transcript, thereby inhibiting its translation process. The activation of the MYEOV gene can be achieved through two pathways: 1. By combining the MYEOV gene with the 5’ intronic Em gene located between the IGH joining and switch sequences, and simultaneously activating it with the CCND1 gene; 2. Through the IGH enhancer in the 3’ regulatory region (RR) downstream of the constant region gene (5).

The MYEOV gene is involved in the enrichment of genes associated with intercellular adhesion, the G1/S transition of the mitotic cell cycle, angiogenesis regulation, and various other cellular processes. These differentially expressed genes primarily possess a range of molecular functions, including cadherin binding and protein binding capabilities (9). Through Gene Set Enrichment Analysis (GSEA) of MYEOV’s functions, it has been found that MYEOV can enrich the “RIG-I-like receptor signaling pathway” (10). MYEOV is co-amplified with cyclin D1 and is overexpressed in tumors of multiple organs. The 5’-untranslated region (5’-UTR) of the MYEOV gene is, containing several upstream AUG start codons, and possesses complex secondary structure folding, indicating that its translation may be regulated by internal ribosome entry sites (IRES) (11).

## MYEOV’s embellishment

MYEOV Modification DNA methylation is a form of DNA chemical modification that can alter genetic expression without changing the DNA sequence. DNA methylation refers to the covalent binding of a methyl group to the 5-carbon position of cytosine in CpG dinucleotides under the action of DNA methyltransferases. A substantial body of research indicates that DNA methylation can cause changes in chromatin structure, DNA conformation, DNA stability, and the way DNA interacts with proteins, thereby controlling gene expression (12).

In certain cell lines, the MYEOV gene undergoes amplification without concurrent expression of the MYEOV protein. This phenomenon suggests that the expression of MYEOV may be subject to epigenetic regulation, particularly the influence of DNA methylation. Jia et al. found that the N6-methyladenosine-related gene MYEOV was identified as a prognostic gene for lung squamous cell carcinom (13). Analysis of the TCGA PAAD cohort through the MethHC database revealed that the methylation level of the MYEOV gene promoter region in pancreatic ductal adenocarcinoma (PDAC) tissues is significantly lower than that in normal pancreatic tissues. Zhang et al. identified a total of 118 methylation-driven genes in PDAC. Among them, MYEOV constituted a risk model. Survival analysis showed that the expression of MYEOV and the combined methylation and expression levels of the gene MYEOV could serve as potential biomarkers for predicting the survival of PDAC patients and drug targets (14). Pan et al. used the methylation profiles of TCGA and GSE60645 and found that 54 CpG methylation sites in the gene promoter were significantly associated with the prognosis of LUAD. Among them, 9 genes were significantly correlated with the expression of MYEOV, and MYEOV was abnormally upregulated in LUAD (15). This is consistent with the research conclusions obtained by Xu et al (16). To explore the relationship between MYEOV mRNA expression and promoter methylation status, researchers treated the HPNE cell line with the DNA methylation inhibitor 5-aza-2’-deoxycytidine (5-aza-dC), with or without the addition of Trichostatin A (TSA), a histone deacetylase inhibitor, according to the experimental design. Results from real-time fluorescent quantitative PCR (RT-qPCR) showed that demethylation treatment could restore the expression of MYEOV in the HPNE cell line (17). This indicates that a hypomethylated promoter state may facilitate the transcriptional activation of MYEOV in pancreatic cell lines. Further research reported that after treating seven different esophageal cancer cell lines (KYSE-30, 170, 200, 510, 790, 960, and 1170) with 5-AzaCd, it was found that in one cell line (KYSE-170), the expression level of MYEOV was high, but there was no accompanying DNA amplification. Despite treatment with 5-AzaCd, the expression level of MYEOV in the KYSE-170 cell line changed little. In contrast, the expression of MYEOV was significantly restored in most other cell lines (18). In three cell lines (KYSE-510, 790, and 1170), the restoration of MYEOV expression was particularly notable, and these cell lines all exhibited amplification of the MYEOV gene, with the restored expression levels being consistent with their respective amplification levels (5). Experiments by Coccaro et al. using the demethylating agent 5-AzaCd revealed that the transcription of MYEOV is silenced by methylation within the 11q13 amplicon. The results suggest that DNA methylation may be a mechanism through which genes involved in amplification can escape upregulation. Janssen et al. found that the expression level of MYEOV was inconsistent with the DNA amplification data. A subset of cell lines that showed DNA amplification but did not have high MYEOV expression were treated with the demethylating agent 5-aza-2’-deoxycytidine, and the MYEOV expression in these cells was successfully restored, indicating that MYEOV was transcriptionally silenced by the DNA methylation mechanism in most of the latter cell lines (19). Future research needs to further clarify the role of DNA methylation in gene silencing of different genes, whether within the same amplicon or between different amplicons on different chromosomes (20).

## MYEOV’s 3 ‘region has transcriptional enhancer activity in human cells

Papamichos et al. used computational analysis and demonstrated that the start codon of the short subtype of MYEOV was evolutionarily acquired after the divergence of Catarrhini and Platyrrhini. Throughout the evolutionary process of Catarrhini, MYEOV acquired an ORF that gradually elongated, a translationally regulated upstream ORF that gradually shortened, and selectively spliced mRNA variants. MYEOV obtained the coding potential specific to human proteins through ORF amplification (21). Data analysis of chromatin states from the BLUEPRINT Consortium indicates that in healthy B cells, the entire region of the MYEOV gene is surrounded by an enhancer chromatin state and extends to the 3’ region of MYEOV. This enhancer region is characterized by the presence of histone marks H3K4me1 and H3K27ac (22). Davidson et al., utilizing data from 182 samples of the ENCODE project, which includes primary cells, tissues, and cell line samples, found H3K27ac peaks in the 3’ UTR region of MYEOV in 131 samples, further confirming the enhancer function of this region in humans (23). The study also discovered that in human and mouse cells (which lack ORF but possess enhancer marks), the presumed enhancer of MYEOV-3’ interacts with CCND1/CCND1 in the 3D genome. This suggests that the deep conservation of this enhancer may extend beyond the ORF shared by most primates. The enhancer element provides a promising avenue for exploring the function of the presumed MYEOV 3’ enhancer in carcinogenesis (24). MYEOV is a primate-specific gene with a *de novo* ORF originating from an evolutionarily older enhancer region. This highly conserved presumed enhancer element can regulate CCND1 in humans and mice, offering the possibility of studying the regulatory function of MYEOV in cancer using non-primate animal models (3).

## The role of MYEOV in tumor cells

### Expression of MYEOV in cancer cells

**Figure 1:**
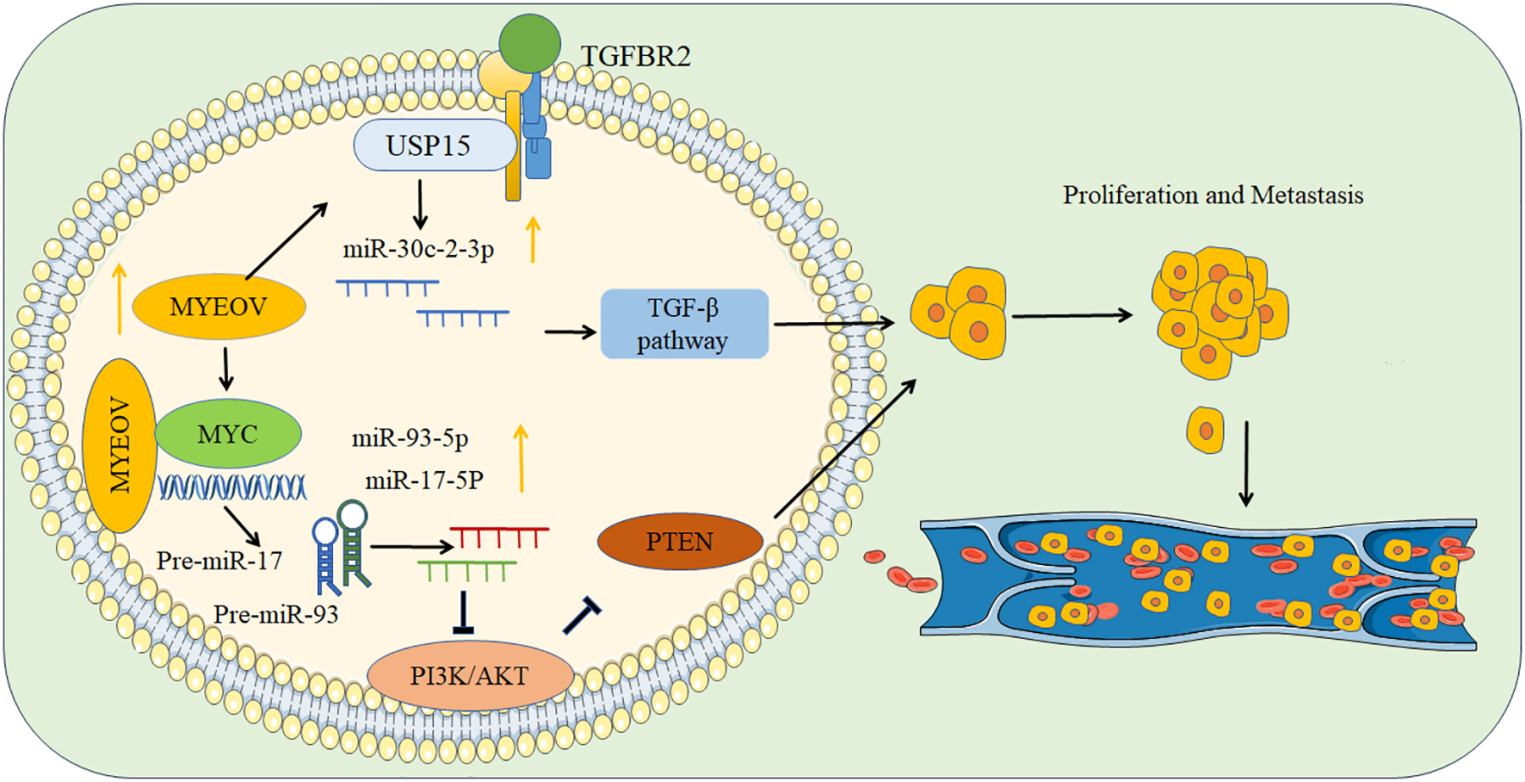
Mechanism of MYEOV promoting tumor proliferation and metastasis.

In the discussion of the occurrence and progression of cancer, the role of the MYEOV protein cannot be overlooked. Kaplan-Meier survival analysis reveals a significant clinical phenomenon: high expression levels of the MYEOV protein are associated with shorter overall survival in cancer patients (4). Through univariate and multivariate Cox regression analysis, a direct link between high MYEOV mRNA expression levels and poor prognosis in the TCGA PAAD cohort was established. **Table 1** summarizes the expression of MYEOV in different types of cancer. In non-small cell lung cancer (NSCLC), the MYEOV gene is upregulated and amplified. **Figure 1** illustrates how MYEOV ceRNA sequesters miR30c-2-3p from its targets TGFBR2 and USP15 mRNAs, leading to the constitutive activation of TGF-β signaling and tumor progression in NSCLC (9). Zhang et al.’s research further confirms the correlation between high expression levels of MYEOV and clinical pathological characteristics in NSCLC. Studies have also reported that MYEOV promotes cell proliferation, survival, and invasion. High MYEOV expression levels are associated with poor prognosis (25). Additionally, the expression levels of MYEOV are negatively correlated with tumor purity and B-cell infiltration levels in LUAD, and positively correlated with the infiltration levels of CD8+ T cells, CD4+ T cells, dendritic cells, and neutrophils in LUSC. Gene set enrichment analysis (GSEA) also reveals the enrichment of high MYEOV expression in specific cancer-related pathways (26). Fang et al.’s research observed that in 107 pairs of TCGA samples, the MYEOV mRNA levels in tumor tissues were more than twice as high as in matched non-cancerous tissues in 92 pairs (86.0%) (9). Studies have also reported that in gastric cancer tissues, the expression levels of MYEOV mRNA are generally higher than in adjacent normal tissues (27). Chen et al. used RT-PCR, *in vitro* cell proliferation assays, pancreatic cancer organoids, and immunohistochemical staining to identify the relationship between pancreatic cancer and MYEOV, and found that knocking down the MYEOV gene could reduce the proliferation of pancreatic cancer cell lines and pancreatic cancer organoids (28). The mRNA expression level of MYEOV is elevated in pancreatic cancer tissues (29). Liang et al. reported high expression of MYEOV in pancreatic ductal adenocarcinoma, and a significant reduction in proliferation of BxPC3 and SUIT2 pancreatic cancer cell lines after knocking down MYEOV with siRNA, indicating a universal role of MYEOV in pancreatic tumors (4). Li et al. analyzed the expression of MYEOV and ANLN genes in clinical specimens, finding that they are moderately positively expressed in pancreatic cancer and share multiple enriched KEGG pathways (30). Studies have also pointed out that the expression of MYEOV is related to the tumor process in PAAD patients, possibly involving the expression of specific transcription factors such as USP22, CDK9, and Foxo1, although this has not been widely reported and requires further research (31). Chen et al. found that the MYEOV transcript may act as a functional RNA molecule in cells, and MYEOV mainly promotes pancreatic cancer through the ceRNA function that is independent of the protein-coding ability. The miRNAs bound to MYEOV-related genes were analyzed. GPRC5A, SERPINB5, EGFR, KRAS, PDCD4, and EIF4G2 all have two miRNAs acting on them, namely hsa-miR-103a-3p and hsa-miR-107. These two miRNAs are also the targets interacting with MYEOV (32). Deng et al. discovered through the TCGA database that MYEOV was significantly associated with the poor outcomes of hepatocellular carcinoma, and MYEOV may be a candidate biomarker for the prognosis of hepatocellular carcinoma (33). In head and neck tumors, overexpression of MYEOV significantly enhances the invasiveness of tumor cells, but in oral *in situ* tumor models, knocking down MYEOV reduces the invasiveness of cancer cells and inhibits tumor growth and metastasis (34). Some studies have reported that in oral squamous cell carcinoma, frequent increases in the 11q13 region are observed in lymph node metastases. There are two distinct amplification cores, which are separated by breakpoints between MYEOV and CCND1 in the 11q13 region. These two amplification cores may have a biological impact on the spread of oral squamous cell carcinoma cells from the primary site to the local lymph nodes (35). Chen et al. reported that MYEOV can promote the proliferation of neuroblastoma-19 cells (36), MYEOV is repeatedly amplified in neuroblastoma, and the survival rate of neuroblastoma patients carrying MYEOV amplification is poor (37). Zhang et al. used Cox regression to screen for prognostic markers of colorectal cancer and found that MYEOV is an independent risk factor for colorectal cancer (38). and Lawlor et al.’s research also found excessive expression of MYEOV in colon cancer (39).

**Table 1:** Expression and function of MYEOV.

Disease Type	MYEOV Expression	Co-Existing Genetic Background	Tumorigenesis and Clinical Outcomes	Ref.
Breast cancer	up	CCND1	Tumor cell growth, Metastasis	(5)
pPRL	up	CCDN1	Tumor cell growth	(8)
Esophageal cancer	up		Tumor cell growth	(18)
NSCLC	up	TGF-β	Tumor cell growth	(9)
NSCLC	up		Short OS	(26)
NSCLC	up		Tumor cell growth and short OS	(25)
Pancreatic cancer	up	PTEN	Tumor cell growth, Metastasis	(60)
Pancreatic cancer	up	SOX9 CCND1	Tumor cell growth, Metastasis	(4)
Pancreatic cancer	up	CXLCL2	Tumor cell growth, Metastasis	(43)
Pancreatic cancer	up	USP22, CDK9, Foxo1	Tumor cell growth, Metastasis	(31)
Pancreatic cancer	up	c-Myc/mTORC1	Tumor cell growth, Metastasis	(17)
Stomach	up		Tumor cell growth, Metastasis	(27)
Head and Neck	up		Tumor cell growth, Metastasis	(34)
Mouth	up		Tumor cell growth, Metastasis	(34)
Neuroblastoma	up	NERGR1	Tumor cell growth	(36)
Colon and Rectum	up	siRNA	Tumor cell growth, Metastasis	(40)
Colon and Rectum	up	COX/pge2	Tumor cell growth	(39)
LUAD	up	GPX4 SMPD1	Short OS	(51)
MMC	up		Tumor cell growth	(42)

Knocking down MYEOV with siRNA resulted in a 48% reduction in cell proliferation and a 36% reduction in cell invasion (40). In a clinical study of 178 PDAC patients, the prognosis of patients with high MYEOV expression was significantly worse than those with low expression (P < 0.05). After the expression of MYEOV increased in PDAC, analysis in multiple PDAC cell lines and normal HPDE cell lines showed that the expression levels of MYEOV in cancer cells increased. Knocking down MYEOV can inhibit the migration and invasion of PANC1 and Capan2 cells. *In vivo*, downregulating the MYEOV gene inhibits the metastasis of PANC1 cells to the liver. In ESC cell lines, MYEOV is co-amplified with CCND1 in the primary tumor (41). Moreaux et al.’s research reported that MYEOV expression in multiple myeloma cells (MMC) can predict patient survival, and knocking out MYEOV can significantly reduce the growth of MMC (42). Chemokines play a key role in the recruitment of immune cells to tumor sites. Downregulating MYEOV can inhibit the expression of CXCL2 in pancreatic cancer cells, and the CXC chemokine CXCL2 has strong neutrophil chemotactic activity. Inhibiting the MYEOV gene may potentially reduce cancer cell metastasis and suppress the immune-suppressing ability of cancer cells (43). A genomic analysis was performed on Japanese patients with stage I esophageal squamous cell carcinoma, and a marginal association was found between MYEOV genomic alterations and progression - free survival (P <.05). This paves the way for the development of new potential treatment methods for patients with esophageal squamous cell carcinoma (44). Luo et al. used the TCGA database and found that MYEOV is a prognostic gene for uveal melanoma. It can accurately identify the prognosis of patients and has a close interaction with neutrophils in the tumor environment (45).

### Expression of MYEOV in non-tumor cells

Studies have reported that in differentiated corneal epithelial cells, MYEOV can reduce the expression levels of 5-hydroxymethylcytosine (5hmC). Through reverse transcription polymerase chain reaction (RT-PCR) technology, it has been found that in classified MUC16-positive cells, MYEOV is significantly overexpressed, and its protein’s *in situ* expression is detected only in the apical epithelial layer of the cornea. These findings suggest that MYEOV is a novel epigenetic regulatory marker gene in the process of corneal epithelial differentiation (46). Zinner syndrome is a rare congenital malformation of the male urogenital system, characterized by a triad: seminal vesicle cyst, unilateral renal hypoplasia, and ipsilateral ejaculatory duct obstruction. A study has reported a patient with seminal vesicle cyst. Whole exome sequencing of his blood and tissue samples revealed the overexpression of MYEOV, and MYEOV may be a potential mutant gene associated with Zinner syndrome (47). MYEOV was also found to be the gene that affects the five-generation Turkish family with hereditary hearing impairment (48). MYEOV has been confirmed to be associated with the waist circumference of Arab obese patients (49).

### MYEOV’s upstream regulator

At the protein synthesis level, the expression of MYEOV is strictly regulated by upstream open reading frames (uORFs). The second pseudo start codon of MYEOV generates a long and weak Kozak sequence, which could significantly affect its translation efficiency (11). Wang et al. used the bioinformatics software TargetScan 7.1 to predict potential target genes of miR-490-5p and found that MYEOV contains a complementary binding site for miR-490-5p. Further luciferase reporter gene assay results indicated that miR-490-5p significantly reduced the relative activity of luciferase in SH-SY5Y and SK-NSH cells transfected with wild-type MYEOV, while having no effect on mutant MYEOV, confirming that miR-490-5p can directly bind to the 3’ untranslated region (3’ UTR) of MYEOV, thereby downregulating the expression of MYEOV (50). Luo et al. reported that in lung adenocarcinoma (LUAD), the SMPD1-induced autophagic degradation mechanism of GPX4 plays a key role in triggering ferroptosis after MYEOV knockout. In LUAD, GPX4 induces the upregulation of MYEOV expression, inhibits cellular ferroptosis, and leads to a decrease in the survival rate of LUAD patients (51). Liang et al.’s research showed that in pancreatic cancer, HES1 is necessary for the oncogenic phenotype mediated by MYEOV; *in vitro* cell line studies revealed that knockdown of HES1 reduced the migratory and invasive potential of tumor cells overexpressing MYEOV (39).

### MYEOV and acidosis

Tumor microenvironment acidosis (TME-A) plays a significant role in tumor immune dysfunction and tumor progression (52). Studies have indicated that MYEOV is a key gene associated with acidosis markers, with elevated expression levels in pancreatic cancer (PC) tumor tissues compared to normal tissues, and this increased expression is closely related to poor clinical outcomes in patients with pancreatic cancer (53).

### MYEOV and glycolysis

The increased metabolic rate of cancer cells leads to intensified glycolysis. Glycolysis is one of the important metabolic pathways in cancer cells, primarily due to the acidic substances produced by cancer cells or the increase in glycolysis through the gluconeogenesis pathway. Glycolysis is usually associated with factors such as cell proliferation, tumor metastasis, and tumor angiogenesis (54). Research by Tang et al. has shown that high expression of MYEOV is significantly correlated with a low survival rate in patients with Pancreatic Ductal Adenocarcinoma (PDAC), with high expression of MYEOV promoting glycolysis in PDAC tumor cells. MYEOV acts as an oncogene in PDAC and can therefore serve as a prognostic biomarker for PDAC patients (41).

### MYEOV died with iron cells

The concept of ferroptosis was first introduced by Dr. Brent R. Stockwell from Columbia University in 2012, and it is a novel form of regulated cell death that is iron-dependent and distinct from traditional cell death mechanisms such as apoptosis, necrosis, and autophagy (55). The core mechanism of ferroptosis lies in the depletion of glutathione (GSH) and the decline in the activity of glutathione peroxidase 4 (GPX4), which prevents the metabolism of lipid peroxides through the glutathione reduction reaction catalyzed by GPX4. Subsequently, ferrous iron ions catalyze lipid peroxidation to produce reactive oxygen species (ROS), thereby triggering ferroptosis and causing cell death (56).

The knockout of the MYEOV gene can inhibit cell proliferation *in vitro* and tumor growth *in vivo*. Moreover, the knockdown of MYEOV induces a significant ferroptotic phenotype, characterized by increased levels of intracellular ferrous ions, reactive oxygen species, and lipid peroxidation, as well as changes in the expression of ferroptosis marker proteins such as SLC7A11, GPX4, FTH1, and ACSL4 (51). In contrast, the overexpression of MYEOV accelerates cell proliferation and suppresses the occurrence of ferroptosis. Tang et al. found that MYEOV showed a trend of high expression in NSCLC specimens with KRAS mutations. MYEOV silencing effectively inhibited the malignant phenotype of NSCLC cells carrying KRAS mutations and promoted ferroptosis (57). As an oncogene that inhibits ferroptosis, MYEOV promotes the proliferation of lung adenocarcinoma (LUAD) and shortens patient survival, potentially making it a promising therapeutic target.

### The relationship between MYEOV and folate metabolism

The one-carbon metabolism pathway, composed of the folate and methionine metabolic pathways, involves a set of key genes in the mitochondrial folate metabolic pathway, which are typically expressed at low levels or not expressed at all in normal adult tissues, but are characterized by high expression in cancer. Genes closely related to the folate cycle include MTHFD1, MTHFD2, and MTHFD1L, among others (58). In cancer cells, the activation of c-Myc and mTORC1 alters cellular metabolic pathways that promote tumor growth and survival. Research by Tange et al. has reported that knocking down MYEOV can downregulate the expression of metabolic genes that are upregulated in tumors (17). Although Tang et al. have reported that MYEOV downregulates glycolytic genes, research findings also indicate that other metabolic genes are affected by MYEOV. MTHFD2, a key enzyme in this pathway with both dehydrogenase and cyclohydrolase enzymatic activities, is reportedly highly expressed at the protein level in various human tumors, including pancreatic cancer, and is negatively correlated with survival rates (53). RNA-Seq results from Tange et al. show that after MYEOV knockout, genes involved in the folate cycle, such as MTHFD1, MTHFD2, and MTHFD1L, are significantly reduced. Similar results were observed in qPCR experiments conducted in other pancreatic cancer cell lines, BxPC-3 and Panc-1.

### MYEOV controls tumor growth and metastasis by regulating miRNA

MicroRNAs (miRNAs) are a class of non-coding single-stranded RNA molecules encoded by endogenous genes, approximately 22 nucleotides in length, and they play a crucial role in the post-transcriptional regulation of gene expression in both animals and plants. miRNA genes are primarily distributed throughout the genome as single copies, multiple copies, or in gene clusters. miRNAs exert precise control over the expression of target genes by degrading target mRNAs or inhibiting their translation process. As key genetic regulatory elements, miRNAs are involved in the regulation of tumorigenesis and the development of cancer (59).

MYEOV is closely associated with the regulation of microRNAs miR-17-5p and miR-93-5p and may interact with the MYC protein. The absence of MYEOV disrupts the transcriptional mechanism of miRNAs, leading to the abnormal expression of miRNAs in tumor cells. In pancreatic ductal adenocarcinoma (PDAC), the knockdown of MYEOV reduces the expression levels of miR-17-5p and miR-93-5p. MYEOV targets the PTEN protein, thereby regulating the PI3K/Akt signaling pathway and affecting the expression of miR-17/93-5p, which in turn participates in the regulation of PDAC cell proliferation, invasion, and migration processes. The downregulation of either miR-17-5p or miR-93-5p can significantly inhibit cell proliferation, invasion, and migration, but inhibiting the expression of these two miRNAs does not affect the expression of MYEOV (60). Researchers used anti-Flag antibody for immunoprecipitation experiments to extract potential interacting proteins from PANC1 and Capan2 cells transfected with Flag-MYEOV or control vectors, and then identified the proteins by mass spectrometry. The study found that SOX9, HSPA8, CSE1L, and DOCK4 are high-scoring candidate genes that may interact with MYEOV in PANC1 and Capan2 cells. Further co-immunoprecipitation (Co-IP) experiments confirmed that only SOX9 binds to MYEOV and is transported into the nucleus together with it. MYEOV and SOX9 are mainly localized in the nucleus of PDAC cells, and although MYEOV lacks a classical nuclear localization signal (NLS), the mechanism of its nuclear import still needs to be further explored. MYEOV can enhance the DNA-binding activity of SOX9 in pancreatic cancer tissue, thereby promoting the progression of pancreatic cancer. In non-small cell lung cancer, MYEOV transcripts bind to mRNA, enhancing the repressive activity of the TGF-β signal, ultimately leading to further cancer progression.

### Clinical application of MYEOV

Immunotherapy has shown significant therapeutic effects in various types of cancer, particularly in the application of immune checkpoint inhibitors (61). MYEOV, as a potential therapeutic target, currently does not have any new drugs targeting this molecule or its related oncogenic mutation resistance that have been developed or approved for clinical use. In the United States, there was a clinical trial targeting MYEOV—Phase II study of bevacizumab and bortezomib in patients with relapsed/refractory multiple myeloma—which was terminated due to insufficient recruitment. MYEOV is considered an independent prognostic factor for several types of human cancer, including pancreatic cancer, and a “viable” therapeutic target. Elevated expression levels of MYEOV appear to be a potential predictive biomarker for anti-MYEOV therapy in several cancer types.

## Discussion and prospect

In this review, we have delved into the multifaceted roles of MYEOV in the tumor microenvironment and highlighted its significant functions in tumorigenesis. By conducting a comprehensive analysis of the expression patterns of MYEOV and their correlations with patient prognosis, tumor-infiltrating immune cells, and the expression of immune checkpoint genes, we have further anticipated the potential application prospects of MYEOV in tumor immunotherapy. Given the current insufficient understanding of the role of MYEOV in tumor drug resistance and immune status alterations, it is necessary to conduct more preclinical and clinical trial studies to obtain more precise information.
